# Antibiotics: Conventional Therapy and Natural Compounds with Antibacterial Activity—A Pharmaco-Toxicological Screening

**DOI:** 10.3390/antibiotics10040401

**Published:** 2021-04-07

**Authors:** Daniel Florin Pancu, Alexandra Scurtu, Ioana Gabriela Macasoi, Daniela Marti, Marius Mioc, Codruta Soica, Dorina Coricovac, Delia Horhat, Marioara Poenaru, Cristina Dehelean

**Affiliations:** 1 Faculty of Medicine, “Victor Babeș” University of Medicine and Pharmacy Timisoara, Eftimie Murgu Square No. 1, 300041 Timisoara, Romania; pancudaniel87@gmail.com (D.F.P.); deliahorhat@yahoo.com (D.H.); marioara.poenaru@gmail.com (M.P.); 2 Faculty of Pharmacy, “Victor Babeș” University of Medicine and Pharmacy Timisoara, Eftimie Murgu Square No. 2, 300041 Timisoara, Romania; alexandra.scurtu@umft.ro (A.S.); marius.mioc@umft.ro (M.M.); codrutasoica@umft.ro (C.S.); dorinacoricovac@umft.ro (D.C.); cadehelean@umft.ro (C.D.); 3 Research Center for Pharmaco-Toxicological Evaluations, Faculty of Pharmacy, “Victor Babes” University of Medicine and Pharmacy Timisoara, Eftimie Murgu Square No. 2, 300041 Timisoara, Romania; 4 Faculty of Medicine, Western University Vasile Goldis Arad, 94 Revolutiei Blvd., 310025 Arad, Romania

**Keywords:** antibiotics, toxicity, antibiotic resistance, classification, natural compounds, antitumor, mechanism of action

## Abstract

Antibiotics are considered as a cornerstone of modern medicine and their discovery offers the resolution to the infectious diseases problem. However, the excessive use of antibiotics worldwide has generated a critical public health issue and the bacterial resistance correlated with antibiotics inefficiency is still unsolved. Finding novel therapeutic approaches to overcome bacterial resistance is imperative, and natural compounds with antibacterial effects could be considered a promising option. The role played by antibiotics in tumorigenesis and their interrelation with the microbiota are still debatable and are far from being elucidated. Thus, the present manuscript offers a global perspective on antibiotics in terms of evolution from a historical perspective with an emphasis on the main classes of antibiotics and their adverse effects. It also highlights the connection between antibiotics and microbiota, focusing on the dual role played by antibiotics in tumorigenesis. In addition, using the natural compounds with antibacterial properties as potential alternatives for the classical antibiotic therapy is discussed.

## 1. Introduction

The discovery of antibiotics in a time when humankind was decimated by different infectious diseases represented a miraculous medical act. Since then, antibiotics have remained in the focus of researchers from different medical fields due to the multiple essential roles played by these compounds in the fight against various pathogens that threaten human health. The development of resistant bacteria to almost all existent antibiotics has become a global threat and an issue of great concern. Moreover, the misuse and the overuse of antibiotics, together with antibiotic-related severe side effects, are clear signals for the imperative need to discover novel therapeutic approaches.

The present manuscript provides a global perspective on antibiotics in terms of evolution from a historical perspective with an emphasis on the up-to-date classification and adverse effects associated with antibiotics therapy. It also highlights the connection between antibiotics and microbiota, focusing on the dual role played by antibiotics in tumorigenesis, with using natural compounds with antibacterial properties as potential alternatives for the classical antibiotic therapy being discussed.

## 2. History of Antibiotics

Bacteria were first identified in the 1670s by Antoni van Leeuwenhoek [1]. Two centuries later, a strong correlation between bacteria and diseases was observed. This discovery attracted interest of the researchers not only to answer some questions about infectious diseases, but also to find a substance that could kill, inhibit or slow down the growth of disease-causing bacteria [2].

Antibiotics are considered one of the most successful discoveries in the history of medicine. For thousands of years, people were helpless in the face of the huge waves of epidemics, such as cholera, smallpox, plague, typhoid fever, malaria, tuberculosis, leprosy and syphilis, since nothing was known about infections, their prevention, antibiotics or vaccines. The situation began to improve along with the discovery of the healing effects of antibiotic-producing microorganisms. The discovery of antibiotics was the key to the continuance of modern medicine. Today, antibiotics, along with extensive knowledge of pathogens, maintaining hygiene measures and the control of infectious diseases, has increased quality of life and life expectancy [3].

There is evidence in Eber’s Papyrus that mushrooms, molds and herbs were used 2000 years ago, without knowing their enormous therapeutic utility, so that, in Egypt, Greece and China, open wounds were treated with poultices of moldy bread [4].

The development of anti-infective drugs was pioneered by Paul Ehrlich, who identified arsphenamine (salvarsan), in 1909, the first sulfa drug which was widely used to treat syphilis just a few months after its discovery [5]. Salvarsan was replaced by the sulfonamide prodrug, Prontosil, discovered by Gerhard Domagk. The bacteriologist continued P. Ehrlich’s work on the study of sulfonamides, drugs that were the first effective, broad-spectrum antimicrobials for clinical use, but which were later replaced by penicillin discovered by Alexander Fleming [6,7]. Thus, A. Fleming revolutionized the antimicrobial treatment by discovering one of the most important drugs of the last century, penicillin, isolated from the cultures of *Penicillium notatum* [8]. In 1945, the dilemma of the β-lactam structure of penicillin was solved, a finding that led to the development of new semisynthetic derivatives able to overcome the resistance to penicillin [9,10].

In 1943, Selman Waksman discovered streptomycin, the first aminoglycoside compound isolated from actinomycetes and the first antibiotic remedy for tuberculosis [11].

The discovery of cephalosporins began in 1945 by the actions of Giuseppe Brotzu, who identified the fungus *Cephalosporium acremonium* from sewage in Sardinia, a city where he noticed that the incidence of typhoid fever was lower than in other areas. Thus, he isolated cephalosporin P and N, antibiotics active on Gram-positive and -negative bacteria, respectively [12].

**Figure 1:**
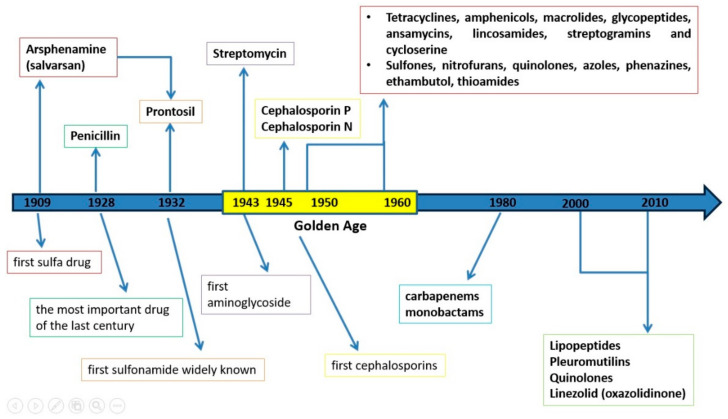
The evolution of antibiotics.

The period between 1940 and 1960 is considered the golden age for antibiotic discovery (Figure 1). During this period, the following were discovered: (i) natural antibiotics isolated from actinomycetes, e.g., aminoglycosides, tetracyclines, amphenicols, macrolides, glycopeptides, ansamycins, lincosamides, streptogramins and cycloserine; (ii) antibiotics of fungal origin, e.g., penicillins and cephalosporins; and (iii) synthetic antibiotics, such as sulfones, nitrofurans, quinolones, azoles, phenazines, ethambutol and thioamides. The vast majority of these antibiotics are still used as therapeutics, but, due to increased antimicrobial resistance, their efficacy has decreased [9].

In the 1980s, other β-lactam anti-infective chemotherapeutics were identified, such as carbapenems and monobactams, broad-spectrum antibiotics [13]. Since 2000, new antibiotics have been launched worldwide, including natural product- and synthetically-derived compounds. The first natural product-derived antibiotics approved for human use were daptomycin and retapamulin, members of the lipopeptide and pleuromutilin classes. Within the synthetically derived antibiotics there was minimal diversity; the novel antibiotics belonged to the quinolone and oxazolidinone (linezolid—the first member synthetized) classes [14]. The timeline of antibiotic development is shown in Figure 1.

Bacteria changed over time in response to the environmental challenges. Nowadays, antibiotics are very frequently prescribed and consequently we are confronting an increase of antibiotic resistant bacteria. A clear example is represented by *Staphylococcus aureus*, which was initially sensitive to penicillin, but, in time, the drug became ineffective, since the bacterial strains developed an enzyme capable of blocking penicillin’s effect. To counteract the antibiotic resistance, a novel form of penicillin immune to the enzyme’s effects has been developed, but, after a few years, the bacteria adapted and became resistant to the new drug. Thus, over the years, an increased number of antibiotic resistant bacteria has been recorded [15].

Natural products and their derivatives continue to play a dominant role in the development of drugs used for the treatment of human diseases. However, the current situation is alarming: resistance to antibiotic continues to increase and poses a global threat to human health. Therefore, novel classes of antibiotics and chemical changes of known antibiotic scaffolds are needed [3,16].

Over the years, it has been observed that microorganisms generated 40,000 antibiotics and “higher” organisms including plants and animals produced approximatively 25,000 antibiotics. The total estimated number of natural antibiotics varies from about 65,000 to 70,000 and the number of semisynthetic and synthetic compounds derived from them can be estimated at about 100,000. However, out of this high number of compounds, only a few hundred are used in clinical practice [16].

## 3. Classification of Antibiotics

According to data in the literature, antibacterial agents can be classified into several major groups, on the basis of the following criteria: source, chemical structure, mechanism of action, type of action and spectrum of activity [17].

### 3.1. Antibiotics’ Classification by the Nature of the Source

Based on the nature of the source, antibiotics can be grouped into the following: (i) natural compounds obtained from microorganisms; (ii) semi-synthetic members that are structurally modified natural products; and (iii) synthetic products. Natural antibiotics (benzylpenicillin, cephalosporins and gentamicin) present a critical inconvenience, high toxicity, whereas semi-synthetic (ampicillin and amikacin) and synthetic antibiotics (moxifloxacin and norfloxacin) ones exhibit an augmented therapeutical effect and a lower toxicity as compared to natural antibiotics [18].

### 3.2. Antibiotics’ Classification Based on Chemical Structure

**Figure 2:**
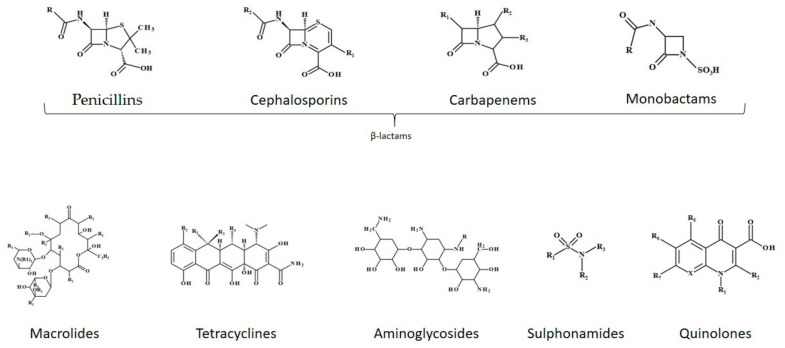
Main classes of antibiotics and their general chemical structure.

The family of antibiotics comprises various members that present different chemical structures as well as unique therapeutic behavior related to structure, therefore chemical structure is considered a reliable criterion for antibiotics classification. On this basis, antibiotics have been categorized in the following classes: β-lactams, macrolides, tetracyclines, aminoglycosides, sulfonamides and quinolones (see Figure 2) [19].

β-lactam antibiotics are one of the most popular classes of antibiotics, having specific signature of the presence of β-lactam ring with the differences between the members of the class being made by the attached side chain or additional cycles. The representatives of this class are penicillins (presenting a thiazolidine ring and a side-chain that differs for each member), cephalosporins (possessing a dihydrothiazine ring and two side chains), carbapenems (a thiazolidine ring structure slightly different from penicillins) and monobactams (with β-lactam ring and no adjacent ring) [20,21]. Sulfonamides are a group of synthetic compounds with great medicinal importance that contain the sulfonamide chemical group (R-SO-NR R) in their structures. Tetracyclines are compounds with a linearly fused tetracyclic nucleus to which a variety of chemical groups are attached. The first group of molecules of the class were obtained from *Streptomyces aureofaciens* and *Streptomyces rimosus*, respectively, but the most recently discovered molecules are semisynthetic [22]. Macrolides were originally isolated from *Streptomyces* species. Macrolides are antibiotics that consist of a macrocyclic lactone ring, usually 14-, 15- or 16-membered-ring compounds to which various amino sugars are attached [23,24]. Quinolones are potent synthetic antibacterial agents, molecules structurally derived from the heterobicyclic aromatic compound quinoline. Substitution on certain parts of the quinolone nucleus may increase potency of the molecules, namely in positions C1 (e.g., difluorophenyl or cyclopropyl), C6 (a fluorine—fluoroquinolones) and C8 (a halogen, methoxy or fused third ring) [25,26].

The general chemical structures of the main classes of antibiotics are presented in Figure 2.

### 3.3. Antibiotics’ Classification by the Mechanism of Action

The structural diversity of the antibiotics is directly correlated to different mechanisms of action. Previous studies defined as main targets of antibiotics within the bacteria, the following: cell wall synthesis, protein synthesis, cell membrane function and nucleic acid synthesis, processes that play key roles in bacterial growth [2]. On this basis, the antibiotics can be classified by the mechanism of action as inhibitors of cell wall synthesis, protein synthesis, cell membrane function and nucleic acid synthesis (see Table 1). Another described antibiotic mechanism of action consists of blockage of key metabolic pathways [27,28].

**Figure 3:**
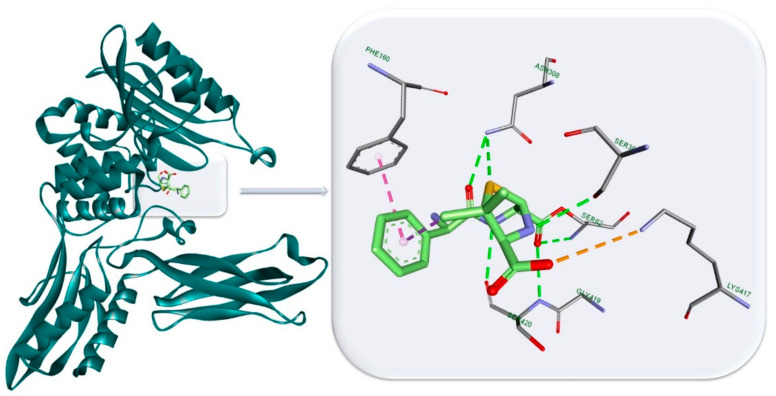
Penicillin binding protein 4 (PBP4) from *E. coli* in complex with ampicillin (light green): HBs (Ser 30, Ser 62, Ser 420 and Asn 308) are depicted as green dotted lines, Pi-hydrophobic interactions (Phe 160) are depicted as purple dotted lines and electrostatic interactions (Lys 417) as orange dotted lines. The figure was built with Biovia Discovery Studio 4.1 using the PDB entry 2EX6 [29].

Inhibition of the bacterial cell wall synthesis represents an important step in arresting the bacterial growth by suppressing the formation of the peptidoglycan layer. The β-lactam family exerts its particular bactericidal activity through binding to the bacterial membrane receptors known as penicillin-binding proteins (PBP), due to their structural similarity with the endogenous PBP substrate, D-alanyl-D-alanine. At the active site, β-lactams acetylate the serine residues, rendering the enzyme unable to further bind with its natural substrate. The penam ring plays an important role in forming key hydrogen bonds (HBs) within the PBP’s binding site [29]. Ampicillin, for example, binds particularly well within the PBP’s binding site, forming multiple HBs with Ser and Asp residues through carboxylic and amide O atoms as well as heterocyclic N and S. Ampicillin binding interactions complexed with PBP are depicted in Figure 3 [29]. β-lactams together with other antibiotic classes (bacitracin, vancomycin, teicoplanin, novobiocin, etc.) are known to act as inhibitors of bacterial cell wall synthesis leading to cell envelope injury and loss of structural integrity [2,30].

**Figure 4:**
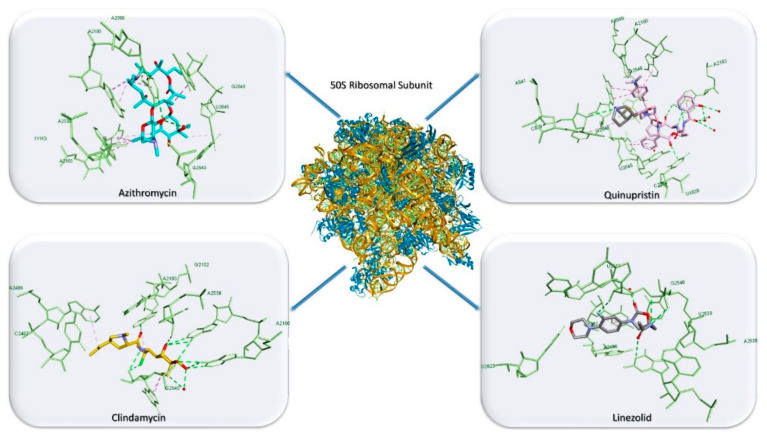
Molecular interactions of 50S ribosomal subunit targeting antibiotics, azithromycin (light blue), clindamycin (orange), quinupristin (pink) and linezolid (grey): HB are depicted as green dotted lines and hydrophobic interactions are depicted as purple dotted lines. The figure was built with Biovia Discovery Studio 4.1 using the PDB entries 1YHQ, 1YJW, 3CPW and 1YJN [31,32].

Protein synthesis is considered a vital function for cells’ survival in both bacterial and mammalian cells, a role that makes it a reliable target for antibiotic activity. Protein synthesis in bacterial cells involves multiple steps: initiation and elongation that comprises the entry of aminoacyl tRNA, proofreading, peptidyl transfer, the translocation of the ribosomes and termination [2]. Any of these steps could be impaired by antibiotics via binding to the 30S or 50S ribosomal subunits depending on the type of antibiotic. The classes of antibiotics that target bacterial protein synthesis are aminoglycosides, macrolides, tetracyclines, streptogramins, chloramphenicol, clindamycin, etc. (Table 1). Most class representatives that bind to the large 50S ribosomal subunit occupy the nascent peptide tunnel site inhibiting new peptide formation. One particular characteristic of these compounds (azithromycin, clindamycin and quinupristin) consists of binding the A2099 residue (G2099 mutated) through a HB. Even if G2099A mutations do not affect the ability of HB formation, they account for loss of drug binding potency and are one of the incriminated factors for drug resistance development [31]. Figure 4 depicts binding interactions of azithromycin, clindamycin, quinupristin and linezolid in complex with the 50S large ribosomal subunit [31,32]. 

**Figure 5:**
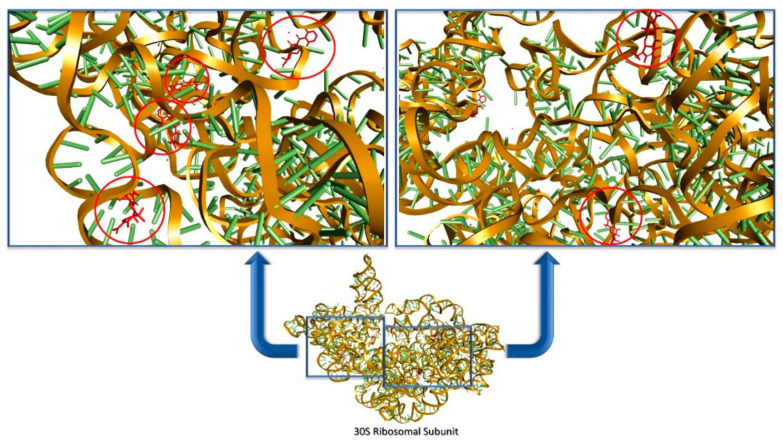
Small 30S ribosomal subunit from *T. thermophilus* in complex with tetracycline (red); zoomed image shows six different binding sites of tetracycline within the ribosomal subunit’s structure. The figure was built with Biovia Discovery Studio 4.1 using the PDB entry 1I97 [36].

Other classes of antibiotics such as aminoglycosides or tetracyclines share a similar protein synthesis inhibition mechanism of action. However, these drugs inhibit translation by targeting the small 30S ribosomal subunit’s decoding center [33]. Aminoglycosides such as neomycin and paromomycin, when bound to the 30S ribosomal subunit, trigger a conformational change in the ribosomal unit’s A-site that leads to codon misreading and mRNA mistranslation [34]. Tetracyclines occupy the same active site within the A site. This complex formation inhibits the protrusion of acyl-tRNA into the active site which can no longer be recognized by the mRNA codon. The process incapacitates the ribosome for protein synthesis [35]. However, crystallographic data show that tetracycline not only binds in the decoding site but is also located in five supplementary binding pockets, within the 30S subunit [36] (Figure 5).

**Figure 6:**
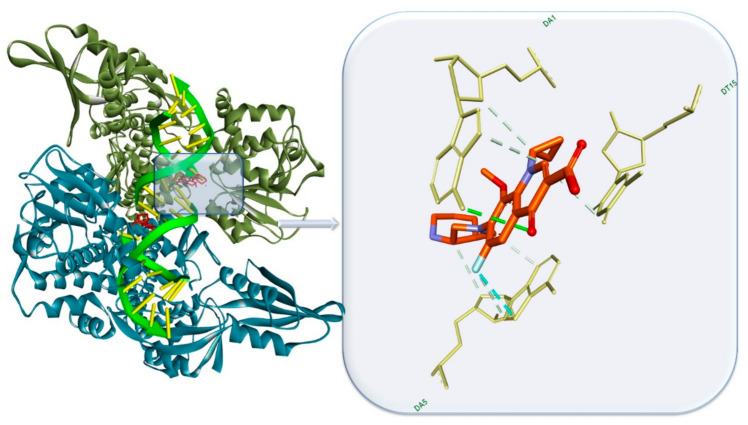
Topoisomerase IV from *S. pneumoniae* in complex with moxifloxacin (red) and DNA (light green): classical HB are depicted as green dotted lines (DA1), C-H HBs are depicted as light green dotted lines and halogen interactions are depicted as blue dotted lines (DA5); hydrophobic interactions were omitted for better picture clarity. The figure was built with Biovia Discovery Studio 4.1 using the PDB entry 4Z3O.

Nucleic acid synthesis arrest in bacterial cells occurs via the inhibition of the enzymes’ activity responsible for DNA or RNA synthesis. The antibiotics that trigger inhibition of RNA synthesis disrupt the bacterial transcription process, leading to impaired cell viability. Rifamycin class and fidaxomicin/lipiarmycin are the antibiotics known as inhibitors of the bacterial RNA polymerase [2,37]. The DNA inhibitors exert their effect by suppressing the DNA synthesis within bacterial cells by interfering with the activity of the type II topoisomerase enzymes: DNA gyrase and DNA topoisomerase IV [38]. The antibiotics recognized as DNA inhibitors are quinolones and metronidazole (Table 1). Figure 5 illustrates the moxifloxacin’s mechanism of action in *S. pneumoniae* by interfering with the topoisomerase IV. The structure interacts with two adenine residues (Figure 6) through a classical HB formed between the drug’s oxo group and the nucleotide amino group and a halogen bond between the florine and the heterocyclic N atom. 

**Figure 7:**
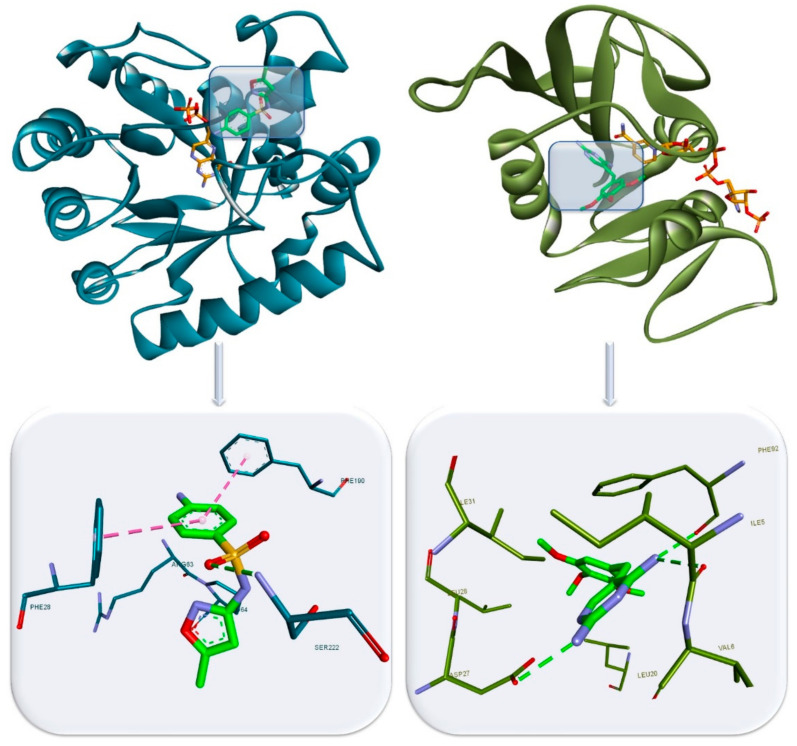
Dihydrofolate reductase chain A (right) from *S. aureus* in complex with trimethoprim (green) and NADPH (orange) and dihydropteroate synthase chain B (left) from Y. pestis in complex with sulfamethoxazole (green) and 6-hydroxymethylpterin-diphosphate (orange). The figure was built with Biovia Discovery Studio 4.1 using the PDB entries 3TZF and 2W9S [40,41].

Another attractive target for antibiotics in bacteria is represented by folate metabolism, mainly the impact on dihydrofolate reductase (DHFR), a key enzyme involved in multiple pathways as thymidylate synthesis, DNA replication and cell survival. A well-known selective inhibitor of the bacterial DHFR is trimethoprim, but other compounds are also investigated in this regard [39]. Trimethoprim binds to the active site of DHFR through HBs formed by the amino groups with the adjacent amino acid residues (Figure 7) [40]. Sulfonamides also act as folate antimetabolites by inhibiting dihydropteroate synthase (DHPS). A key structural feature of the sulfa drug is the sulfonamide group which aids in binding the structure through a HB. The phenyl stabilizes the structure within the active site by π–π stacking with adjacent Phe residues. The binding interactions of a sulfamethoxazole complexed with DHPS are depicted in Figure 7 [41]. 

### 3.4. Antibiotics’ Classification by the Type of Pharmacological Effects

The pharmacological effects of antibiotics—bactericidal or bacteriostatic—are used as a criterion to classify these compounds. Bactericidal compounds trigger bacterial cell death via inhibiting cell wall synthesis, cell membrane function or protein synthesis and. This category includes β-lactams, aminoglycosides, glycopeptides, ansamycins, quinolones, streptogramins, lipopeptides and macrolides [2]. As regards the bacteriostatic agents, their effect consists of halting the bacterial cellular activity and growth without triggering cell death [42]. This class includes sulfonamides, tetracyclines, chloramphenicol, oxazolidinones and macrolides. 

### 3.5. Antibiotics’ Classification Based on the Spectrum of Activity

**Figure 8:**
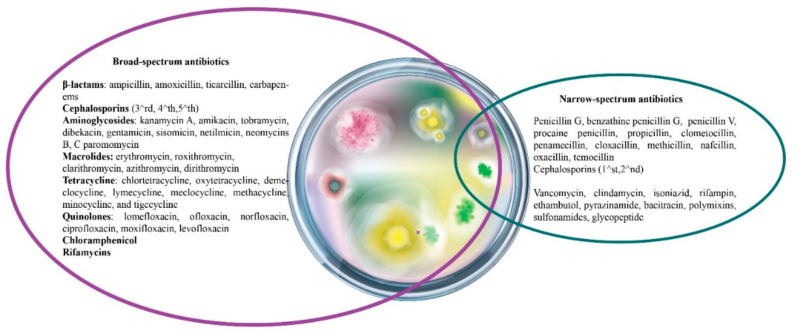
Graphical representation of broad- and narrow-spectrum antibiotics.

Dependent on their spectrum of activity, antibiotics can be grouped into broad- and narrow-spectrum antibiotics. The broad-spectrum compounds are effective against a wide variety of pathogenic bacteria (both Gram-positive and -negative bacteria), whereas narrow-spectrum agents exert their effect only against one type of pathogenic bacteria (Gram-positive or Gram-negative bacteria). According to the existent experimental evidence, narrow-spectrum antibiotics are considered ideal antibacterial agents and are preferred to broad-spectrum ones, due to their specificity and reduced bacterial resistance development [43]. The main representatives of both classes are depicted in Figure 8. 

## 4. Antibiotics’ Toxicity

Antibiotics play an important role in increasing life expectancy by reducing the morbidity and mortality related to infectious diseases [44]. The success of penicillin for the treatment of pneumonia, streptomycin for tuberculosis and chloramphenicol for typhoid fever was monumental; thus, their side effects remained almost invisible [45].

Antibiotics occupy the second position as cause of drug-related side effects and are one of the classes of drugs most frequently associated with medical malpractice [46].

Antibiotics’ toxicity was observed, in particular, in patients with renal or hepatic dysfunction, as well as after administration of high doses or long-term treatments with high doses of antibiotics. Most antibiotics-related adverse effects are unpredictable because of their idiosyncratic nature, due to innate individual hypersensitivity; thus, it is necessary to understand the basic mechanisms involved in their occurrence and identify the individual contributory factors [47].

Antibiotics, as with any class of drugs, have general side effects as well as specific ones for each member, so all these aspects must be considered to prevent toxicity in patients [48].

The most common side effects described after antibiotic treatment are hypersensitivity, hematological reactions and neurological, gastrointestinal, renal, cardiac, pulmonary and hepatic adverse effects.

### 4.1. Hypersensitivity Adverse Effects

Drug fever is one of the most common antibiotic-mediated hypersensitivity adverse effects, which may occur with any antibiotic but is particularly common to β-lactams and some sulfonamides. Drug fever is a febrile response to medication, characterized by transient elevations of the serum transaminases, temperatures ≥ 38.8 °C and relative bradycardia, symptoms that disappear after discontinuation of the incriminated drug [49,50].

Drug-induced rash is a hypersensitivity reaction with cutaneous manifestations that may be caused particularly by β-lactams or trimethoprim-sulfamethoxazole. This type of reaction can appear either on certain parts of the body or on the whole body and the spectrum of dermal manifestations range from maculopapular eruptions to Stevens–Johnson syndrome [51,52,53].

Another hypersensitivity reaction associated with antibiotic treatment is the photosensitivity commonly observed for tontetracycline, sparfloxacin, lomefloxacin and clinafloxacin. This side effect has been sporadically noticed for methacycline and minocycline as well as other fluoroquinolones. Phototoxicity is a dose-dependent phenomenon that requires exposure to direct or indirect ultraviolet light, thus patients are advised to use sunscreen or avoid direct sunlight for at least one week after therapy. It was specified that photosensitivity occurs as a result of fluoroquinolone photodegradation and the molecule’s ability to generate free oxygen radicals that can attack cellular lipid membranes and initiate inflammatory processes [54,55].

Among antibiotics, penicillins are most frequently associated with anaphylactoid reactions. Although there is a strong structural similarity between penicillins and the class of monobactams and carbapenems, their representatives, such as aztreonam and meropenem, can be used in patients with anaphylactic reactions to penicillin. Studies have shown less cross-reactivity between these molecules and penicillins [56,57].

Furthermore, patients with a history of penicillin allergy can tolerate penicillins later in life, as approximately 80% of patients lose their sensitivity in 10 years [58].

Hypersensitivity reactions have also been described as side effects of other antibiotic classes such as lincosamides and macrolides. A patient allergic to different classes of drugs may be predisposed to additional allergies with other classes of medications. The incidence of allergy is influenced by the dose administered and specific disease- and patient-related factors [59].

### 4.2. Hematologic Adverse Effects

Hematologic adverse effects are specific for a wide variety of antibiotics. β-Lactams and trimethoprim-sulfamethoxazole are the two most common causes of isolated leukopenia or thrombocytopenia [60]. In addition, β-lactams may cause autoimmune hemolytic anemia and trimethoprim-sulfamethoxazole may be associated with folate deficiency, which may result in megaloblastic anemia. Chloramphenicol may induce aplastic anemia, a reaction that is independent of dose and route of administration (it has been observed after oral, rectal, topical and intramuscular administration) [48,61].

Antibiotics can cause nonimmune hemolytic anemia in individuals with glucose-6-phosphate dehydrogenase (G6PD) deficiency. Sulfonamides and chloramphenicol were associated with anemia induced via destruction of erythroid precursors in the bone marrow. In addition, there is evidence that carbenicillin presents a direct toxic effect on bone marrow myeloid precursors leading to severe neutropenia [62].

Antipseudomonal penicillins and cephalosporins, such as cefamandole and cefoperazone, are known to cause hemorrhagic problems. Patients receiving high doses of these antibiotics and those with impaired renal function and poor nutritional status are predisposed to bleeding problems. Identification of the risk factors and a correct adjustment of the dosage will remedy the problem of bleeding diathesis [63,64].

### 4.3. Neurologic Adverse Effects

The toxic impact of antibiotics on the central nervous system is less documented. Many factors related to drug metabolism may increase the susceptibility to neurotoxicity, such as the local blood flow, the rate of absorption of the medication and the status of the blood–brain barrier [65].

Penicillins, including penicillin G, piperacillin, ampicillin and amoxicillin, are de-scribed as neurotoxic antibiotics that may cause a variety of neurotoxic reactions, such as confusion, disorientation, seizure and encephalopathy [66].

The molecules with the highest risk of neurotoxicity in the class of cephalosporins are cefazolin, ceftazidime, cefoperazone and cefepime. The clinical manifestations associated with these antibiotics are lethargy, encephalopathy, myoclonus, seizures, nonconvulsive status epilepticus and coma [67]. The mechanism of cephalosporin-induced neurotoxicity is similar to the one described for penicillins and consists of diminished release of GABA, cytokine release and elevated levels of excitatory amino acids [66,68].

In addition, ciprofloxacin, norfloxacin, ofloxacin, gemifloxacin and levofloxacin are also known to induce neurotoxic adverse effects, e.g., headache, confusion, insomnia, seizures, encephalopathy and myoclonus [69,70]. These antibiotics trigger a dose-dependent effect via the inhibition of GABA-A receptors and activation of excitatory NMDA receptors [71,72].

The most common adverse effect related to aminoglycosides is ototoxicity, as a consequence of an excitotoxic activation of NMDA receptors within the cochlea, which could lead to oxidative stress and cell death [68]. In addition, peripheral neuropathy and encephalopathy have been reported after gentamicin’s administration, whereas, in the case of streptomycin, amikacin, tobramycin and neomycin, neuromuscular blockade is the most common adverse effect in patients with myasthenia gravis [73].

Macrolides including erythromycin, clarithromycin and azithromycin can cause neurotoxicity described as ototoxicity by damage to the cochlea, as well as depression of the central nervous system with confusion or excitation with agitation, insomnia, psychosis and exacerbation of myasthenia gravis [68,74].

### 4.4. Gastrointestinal Adverse Effects

Gastrointestinal adverse effects are ranked as the most frequently described effects after antibiotic therapy and include the following: anorexia, nausea, vomiting, diarrhea, epigastric pain and abdominal cramps. These symptoms are dose-dependent and have been described for all antibiotics, mainly for oral formulations [75]. The macrolides are the least tolerated when administered by the oral route. Clarithromycin is associated with gastric discomfort and metallic taste [76]. The manifestations are attributed to a direct irritative or toxic effect of the antibiotic. A remittance of the gastrointestinal adverse effects can be attained by decreasing the doses of the antibiotic, treating the symptoms and co-administering them with food. However, food may interfere with the absorption of erythromycin, oleandomycin or oral penicillins [75].

Tetracyclines are usually well tolerated when administered orally, with the exception of minocycline and doxycycline that may cause gastrointestinal reactions if taken on an empty stomach, thus their administration should always be with food [48].

Another common gastrointestinal side effect induced by antibiotics is diarrhea, which can involve a variety of mechanisms. *Clostridium difficile* diarrhea is viewed as being an irritative diarrhea, caused by the change in the colonic flora, observed after administration of β-lactams. However, quinolones, doxycycline and meropenem are rarely responsible for *Clostridium difficile* diarrhea. Antibiotic-induced non-*Clostridium difficile* diarrhea has been described for therapy with macrolides, ampicillin, ceftriaxone or trovafloxacin [77,78].

### 4.5. Renal Adverse Effects

Antibiotics-induced nephrotoxicity can be described as glomerular or tubular toxicity. A nephrotoxic effect is mainly associated with multiple dose aminoglycoside therapy. Aminoglycosides exert their toxicity on the tubules of the nephron, and, after intravenous administration, they saturate the tubular cells [48].

Gentamicin is recognized as the most nephrotoxic antibiotic, followed by amikacin and tobramycin. Tubular regeneration and recovery of kidney function are generally complete after approximately 20 days from discontinuation of treatment [79].

Vancomycin, a glycopeptide antibiotic, is used as treatment for patients with multiple comorbidities and infections with resistant pathogens. Multiple randomized clinical trials have reported an increased risk of acute kidney injury associated with vancomycin administration. The onset of kidney damage occurs after a week of therapy with improvement after discontinuation of the medication. Proinflammatory oxidation, mitochondrial dysfunction and cellular apoptosis leading to proximal tubular injury have been described as the mechanisms underlying the nephrotoxic effect of vancomycin [80].

β-Lactams are also recognized nephrotoxins. Acute interstitial nephritis is most often associated with nafcillin and methicillin. Eosinophiluria, accompanied by fever and rash, is a hallmark of antibiotic-induced acute allergic interstitial nephritis [81,82].

### 4.6. Cardiac Adverse Effects

A cardiotoxic side effect of antibiotics is the prolongation of the QT interval that can lead to ventricular arrhythmias (e.g., torsades de pointes). The antibiotics known to generate prolongation of the QT are the macrolides (iv erythromycin, clarithromycin and azithromycin) and some quinolones (levofloxacin and moxifloxacin). The mechanism underlying macrolides and fluoroquinolones’ cardiotoxicity involve the blockage of the rapid component (IKr) on the delayed rectifier potassium current on cell membrane of the cardiac myocytes [83,84].

The cardiotoxicity of aminoglycosides was also evaluated. Adams et al. observed that high concentrations of gentamicin, kanamycin, amikacin and sisomicin reduced isometric contractile tension of electrically driven left atria of guinea pigs. Besides, they discovered that gentamicin not only produced a negative inotropic effect in isolated heart muscle but also decreased contractile responses to several positive inotropic interventions [85].

### 4.7. Pulmonary Adverse Effects

The pulmonary adverse effects related to antibiotic therapy are often caused by nitrofurantoin. Nitrofurantoin-induced pulmonary toxicity can be acute or chronic, with different clinical manifestations. Acute pulmonary reactions result in fever accompanied by pulmonary infiltrates with varying degrees of respiratory insufficiency, pleural effusions and peripheral eosinophilia. Chronic pulmonary reactions are slowly progressive inflammatory processes that can result in pulmonary fibrosis, an irreversible pathology [48].

### 4.8. Hepatic Adverse Effects

Penicillins are a well-recognized cause of liver injury, carbenicillin and oxacillin being the drugs most frequently responsible for antibiotic-induced hepatitis. Elevations in the serum transaminases have been associated with isoniazid administration, as well as trovafloxacin, and they can occur after a single oral or intravenous dose. In addition, trovafloxacin produces fatal hepatic necrosis caused by an idiosyncratic hypersensitivity reaction. Nitrofurantoin administration is one the causes of antimicrobial-induced cholestasis and rarely can determine chronic active hepatitis [48,86].

Among macrolides, erythromycin estolate has been reported to produce subclinical elevation of serum aminotransferases and hepatitis in patients treated for more than two weeks, and some cases of hepatotoxicity have been ascribed to josamycin and roxithromycin [87].

Clinicians should be familiar with the most common adverse effects of the most frequently used antibiotics to minimize the potential for side reactions and eliminate the use of drugs associated with chronic or fatal toxicities.

### 4.9. Accurate Selection of Relevant Criteria to Reduce the Toxicity of Antibiotics

The overuse and misuse of antibiotics led an increase in bacterial resistance paradigms, and approaches that maintain the efficacy of currently used antibiotics are required. The first rule in choosing an appropriate antibiotic therapy should be whether there is an indication for an antimicrobial agent. Signs and symptoms of the infection should be monitored, as well as factors, including age, the patient’s medical history and the presence of comorbidities. Once it is decided that an antibiotic is warranted, the correct use of the agent should be explored, including resistance, benefits, safety and costs [88]. To properly select an antibiotic, randomized and controlled clinical trials provide the highest quality information for making decisions [89].

To improve the patient’s health and reduce unnecessary prescribing, the causative pathogen must be identified and any evidence that a particular antibiotic can lead to cure, as well as the failures associated with the absence of drug treatment, must be considered. If there is substantial resistance to a particular class of antibiotics, it should be replaced with another class of drugs [88]. 

In treating patients with a particular drug, safety comes first. Treatment strategies should be chosen to maximize the efficacy while reducing the side effects. The optimal duration of treatment means the prescription of the selected drug for the shortest period of time necessary for clinical and microbiological efficacy, having the effects of reducing the manifestation of adverse reactions, increasing patient adherence, decreasing resistance and decreasing costs [90].

The choice of an inappropriate therapy or an ineffective antibiotic is associated with increased costs, including the cost of the drug and the increase in the general costs of healthcare due to treatment failures and side effects [91].

Thus, for an appropriate, effective antibiotic treatment, the described criteria must be used, namely evidence-based results, therapeutic benefits, drug safety, short duration of treatment and cost-effectiveness.

### 4.10. Bacterial Resistance to Antibiotics

In the middle of the 20th century, antibiotics were considered miracle drugs, capable of destroying disease-causing pathogens without affecting the host [92]. The basic mechanism by which antibiotics exert their therapeutic effect is complex and consists in the inhibition of the bacterial cell wall synthesis, protein synthesis and deoxyribonucleic and ribonucleic acid synthesis, disorganization of the cell membrane structure or other actions [93]. 

Bacterial resistance became part of the normal cycle of antibiotic development, occurring without exception after their discovery and clinical implementation. There is no class of antibiotics lacking bacterial resistance [94]. 

The increased demand for antibiotics and their widespread and irresponsible use have significantly contributed to the emergence of resistant strains [95]. At the beginning, antibiotic production was directly proportional to the development of resistant strains. Currently, the general approach to fight infections and bacterial resistance is to modify the existing antibiotics [96]. 

Bacterial resistance is developing very rapidly, which is why it has become a major problem for human health. With the improvement of technology, more and more people are aware of the negative effects due to the resistance to available drugs; nevertheless, very few are taking action. In most developing countries, almost all antibiotics are available without a prescription, which is the main factor causing resistance [97]. 

Many organizations such as the World Health Organization (WHO) consider bacterial resistance a major problem and have tried to prevent it from spreading. However, global antibiotic resistance does not show any decline. Antibiotics have played a major role in modern medical development, and, today, antibiotics are essential drugs in all healthcare systems. The successes of modern medicine, such as organ transplantation, cancer therapy, treatment of premature babies or major surgery, are based on antibiotics to control and prevent bacterial infections. If effective global action plans are not adopted, we will face terrible social, medical and economic complications [98].

At the base of bacterial resistance stand four main categories of mechanisms: (i) limitation of drug penetration into the cell; (ii) change of the drug target; (iii) inactivation of the antibiotic; and (iv) activation of the efflux pump. Due to structural differences, Gram-negative and Gram-positive bacteria differ in the mechanism of bacterial resistance. Gram-negative bacteria use all four mechanisms, while Gram-positive bacteria use only two, namely changing the antibiotic target and inactivating the antibiotic [99].

Due to the growing resistance of bacteria to classical antibiotics, interest has increasingly focused on medicinal plants. Pure extracts obtained from various plants can serve as an excellent alternative to classic antibiotics. Numerous metabolites secreted by plants have the ability to kill microorganisms by affecting the host’s cellular processes (immune response, mitosis, apoptosis and signal transduction). For this reason, bacteria are less likely to develop resistance to herbal products [100].

## 5. Natural Compounds with Antibacterial Effect

Antibiotic resistance represents a major health issue at present. In 2013, more than nine million deaths worldwide were due to bacterial infections [101]. In recent years, there has been decreased interest in the field of novel antibiotics development, which could be correlated with the constant increase of antibiotic resistance and the number of deaths [102]. Particular attention should be paid to the plant kingdom, which offers many compounds with antibacterial effects that have been to be effective in the treatment of bacterial infections, without presenting the adverse effects specific for conventional antibiotics.

**Figure 9:**
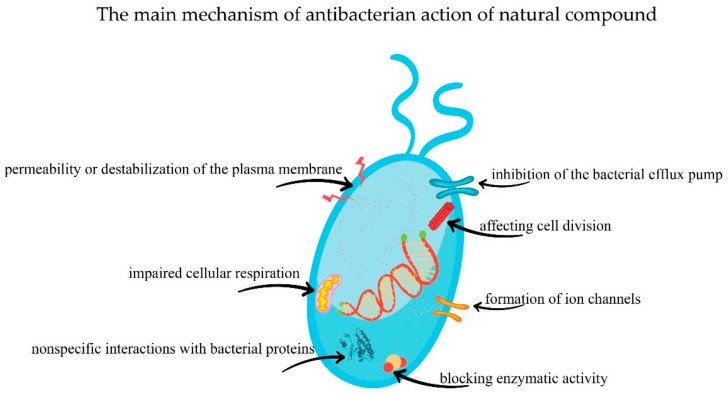
Schematic presentation of the main mechanisms of antibacterial action of plants.

Natural products offer an extraordinary chemical diversity with a plethora of biological effects and thus are the most promising sources for drug discovery and development [103]. The antimicrobial activity of plants depends on the type and amount of constituents (see Figure 9). The most common classes of phytochemicals with antibacterial activity are phenols, polyphenols [104], terpenoids, essential oils [105], alkaloids [106], lectins and polypeptides [107], as well as their mixtures [108]. Between 1986 and 2006, more than 100 antimicrobial drugs were approved for clinical use, 75 being of plant origin [109]. 

### 5.1. Phenols and Polyphenols

Phenolic compounds are secondary metabolites involved in many important plant processes, such as the defense and adaptation mechanism and the pigmentation process. In terms of human health, these compounds have proven to be beneficial in many diseases including cardiovascular disease, cancer and diabetes [110]. 

Due to the differences in the chemical structure of the compounds in this class, the mechanism of antibacterial action is complex, comprising: (i) permeability or instability of the plasma membrane; or (ii) blockage of the extracellular enzymes. Plants rich in these compounds could be considered a reliable alternative to antibiotic resistance since their mechanism of action differs from that of classical antibiotics [111]. 

The antibacterial effects of phenolic compounds have been tested in vitro. In one study conducted on the germs that cause periodontitis, two phenolic compounds (pyrogallol and pyrocatechol) proved to be efficient: pyrogallol had the minimum inhibitory concentration (MIC) value between 2 and 2500 μg/mL, while, for pyrocatechol, the MIC value was between 4 and 312 μg/mL [112]. Thorough studies on Gram-negative and -positive bacteria have shown that pyrogallol is the most active phenolic compound compared to catechol and resorcinol, proving that hydroxyl groups are closely related to the antibacterial activity [113]. The simple phenols’ mechanism of action is not fully elucidated, but studies have shown that antibacterial activity is mainly due to: (i) the interaction with sulfhydryl groups in bacterial enzymes leading to the inhibition of their activity; and (ii) the nonspecific interactions with bacterial proteins [114]. 

Another category of phenolic compounds is represented by the phenolic acids. This category includes caffeic acid, gallic acid and ferulic acid. In a comparative study between caffeic acid and ampicillin, it was observed that caffeic acid exhibited a more pronounced antibacterial activity on *S. aureus* and *E. coli* species [115]. As for gallic acid, it is a polyphenol with intense antibacterial activity, especially on *Campylobacter* species [116]. The antibacterial activity of gallic acid is due to its ability to impair the structure of the cell wall, causing the loss of cellular material [117].

### 5.2. Terpenoids and Essential Oils

Terpenoids are a large group of hydrocarbons. The most common terpenoids found in the plant kingdom are monoterpenes and sesquiterpenes, but diterpenes and triterpenes have also been identified [118]. Regarding the mechanism of action, Griffin et al. [119] showed that terpenoids affect two processes essential for the survival of the bacterial cell: (i) the oxygen absorption; and (ii) the oxidative phosphorylation. Thus, they affect cellular respiration.

The category of monoterpenes includes carvacrol, thymol, menthol and geraniol, which have proven their antibacterial effect on both Gram- and Gram-negative bacteria [118]. Geraniol, unlike the rest of the monoterpenes, has the advantage that it acts evenly on Gram-negative multi-drug resistant (MDR) bacteria. The mechanism of action in MDR bacteria is based on the inhibitory effect on the efflux pump [120]. The monoterpenic alcohols exert a predominantly bactericidal effect [121]. 

Among the sesquiterpenes, farnesol possesses an intense antibacterial activity. Previous studies have shown that farnesol has a moderate effect on *Streptococcus mutans* and *Streptococcus sobrinus* [122] and an intense effect on *S. aureus* and *S. epidermidis* [123]. A considerable number of experiments focused on the combination of farnesol with various antibiotics for the synergistic effect. Two of Masako’s studies tested the combination of farnesol and xylitol. He noted that this association caused an increase in antibacterial activity, and it did not induce the destruction of microbial flora [124]. In another study, Castelo-Branco et al. [125] observed an increment in the potential of action of farnesol on *B. pseudomallai* in combination with amoxicillin, doxycycline and ceftazidime. Recent studies have focused on finding substances that impact on MRSA (methicillin-resistant *Staphylococcus aureus*) and Gram-negative bacteria. The advantage of sesquiterpenes is that they have proven their effectiveness on these bacterial species that threaten human health due to the increased antibiotic resistance [126]. 

Diterpenes are a class of phytocompounds with many therapeutic properties, and, in terms of antimicrobial activity, they have proven their effectiveness on *P. aeruginosa*, *S. aureus*, *E. coli* and *C. albicans.* Hydrogen bonds participate in enhancing antibacterial activity. However, the low water solubility of the compounds is a pharmacokinetic disadvantage. The antibacterial mechanism of action consists of the inhibition of oxygen uptake and oxidative phosphorylation arrest [119]. In addition, diterpenes are active alone or in combination with other antibiotics on MRSA [127]. The class of diterpenes includes salvipisone and aethiopinone, which exert a bactericidal or bacteriostatic effect in MRSA and methicillin-resistant *Staphylococcus epidemidis* (MRSE) [128]. 

Triterpenes consist of six isoprene units [118]. Oleanolic acid, a member of the triterpene class, showed an antibacterial effect on *Mycobacterium tuberculosis* [129]. Besides the oleanolic acid, other triterpenes such as bonianic acid A and B and ursolic acid are active on *Mycobacterium tuberculosis* [130]. In addition, by combining oleanolic acid with gentamicin, an enhancement of the bactericidal activity of gentamicin has been observed [126]. Other triterpenes such as betulinic acid, betulinaldehyde and amyrin have been proved to have an antibacterial effect on MRSA and methicillin-susceptible *Staphylococcus aureus* (MSSA) with a MIC of 2–512 µg/mL [131]. 

Essential oils are volatile substances produced by plants. The therapeutic effects of essential oils are due to the phytocompounds they contain such as terpenes and phenylpropanoids [132]. According to previous studies, essential oils exert an antibacterial effect on Gram-positive bacteria to the detriment of Gram-negative ones [133]. Their antibacterial mechanism of action is complex and includes: (i) destabilization of cellular architecture, impaired membrane integrity and increased permeability [134]; and (ii) reduced membrane potential, proton pump disorder and ATP depletion [135]. 

### 5.3. Alkaloids

The alkaloids represent a group of over 18,000 representatives that possess a heterocyclic structure and one or more nitrogen atoms. Many alkaloids are currently used in therapy: brucine (central nervous system stimulant), ergometrine (oxytocic and vasoconstrictor), quinine (antimalarial), atropine (anticholinergic), etc. [106]. The antibacterial mechanism of action differs depending on the structure of the alkaloid but can be summarized as follows: (i) impaired cell division; (ii) inhibition of respiration and bacterial enzymes’ activity; (iii) damage to the bacterial membrane; and (iv) impact on virulent genes.

The class of alkaloids with antibacterial activity includes pergularinins and thylophorinidines. The mechanism of action is based on the ability of these compounds to inhibit the activity of difolate reductase with negative effects on the nucleic acid synthesis. Another example of an alkaloid with antibacterial action is ugeremine, which acts on the bacterial topoisomerase, exerting an antibacterial effect mainly on the *E. coli* species [136]. Alkyl methyl quinolone alkaloids act by inhibiting cellular respiration. They have a strong and specific action on bacterial respiration in *H. pylori* with a MIC between 0.002 and 0.005 µg/mL [137]. Squalamine affects mainly the cell membrane. Squalamine has a detergent-like effect, causing alteration of the outer membrane of Gram-negative bacteria and depolarization of the membrane of Gram-positive bacteria [138]. In vivo studies described the antibacterial effect of squalamine against *Pseudomonas aeruginosa* [139]. Virstatin is an alkaloid that impairs virulent genes by suppressing ToxT, a regulatory protein involved in the activation of several virulent determinants. Virstatin has been shown to inhibit intestinal colonization with *V. cholerae* [140].

### 5.4. Lectins and Polypeptide

Lectins are a group of natural compounds that contain at least one noncatalytic site for specific and reversible binding to free monosaccharides, oligosaccharides or glycoconjugates. The carbohydrate binding property plays a major role in the immune defense against pathogens, inhibiting the adhesion and migration of microbial cells [141]. 

Two species of red seaweed, *Galaxaura marinata* (GMA) and *Eucheuma serra* (ESA), are known for their lectin content. These types of lectins have proven their antibacterial effect on *Vibrio sp*. Other examples of antibacterial lectins are those from *Bryothamnion triquetrum* (BTL) and *Bryothamnion seaforth* (BSL), which have the property of inhibiting the formation of dental plaque by preventing streptococcal adhesion in enamel films. BTL has a more pronounced effect on *Streptoccocus mitis* and *S. sobrinus*, while BSL has a stronger effect on *S. mutans* [142].

The antibacterial action on Gram-positive and -negative bacteria is explained by the interaction between lectins and the bacterial cell wall through teichoic and teichuronic acids, lipopolysaccharides and peptidoglycans [143]. The lectin extract from *Artocarpus heterophyllus* exerts a potent antibacterial effect on *Bacillus subtilis* and *Pseudomonas aeruginosa* and a moderate antibacterial effect on *Staphylococcus aureus* and *E. coli* [144]. Petnual et al. observed that the lectin isolated from Turmeric longa showed an antibacterial effect on all the bacteria tested, the most sensitive being *Pseudomonas aeruginosa* [145]. 

Plants secrete peptides as secondary metabolites to protect against pathogens. These molecules destroy pathogens by interacting with the membrane phospholipids and alternating membrane permeability [146]. In addition to altering the lipid membrane, peptides have the ability to form ion channels and, implicitly, ion loss such as potassium [147]. 

The most known category of peptides are thionins. In terms of water solubility, thionins are hydrophobic, this property being responsible for their interaction with the cell membrane and causing its lysis [148]. A representative of this class is Cp-thionin II, identified in the plant Vigna unguiculata and known to exhibit antibacterial properties on both Gram-positive and -negative bacteria. Microbiological studies have suggested a minimum inhibitory concentration for Cp-thionin II of 128 µg/mL on *Staphylococcus aureus*, 64 µg/mL on *E. coli* and 42 µg/mL on *Pseudomonas syringae* [149]. Another example of phytocompound with antibacterial action is fabatins that was obtained from the *Vicia faba* plant and shows a broad antibacterial spectrum, against both Gram-negative and -positive bacteria [150]. 

Cyclotides also belong to the family of peptides. Representative antibacterial cyclotides are kalata and circulin A (Cir A) isolated from coffee plants that proved their antimicrobial effect on *Staphylococcus aureus* with a MIC of 0.2 µM. In addition, both are active on Gram-positive and -negative bacteria, but inactive on *Escherichia coli* and *Pseudomonas aeruginosa* [151]. 

### 5.5. Other Phytocompounds with Antibacterial Effect

In addition to the important classes mentioned above, there are other phytocompounds whose antibacterial activity is worth mentioning. The antibacterial activity of thefollowing compounds has been studied and documented: polyamines [152], isothiocyanates [153], glucosides [154] and thiosulfinates [155]. 

Chung-Dar Lu and collaborators observed that polyamines have the property of increasing the sensitivity of *P. aeruginosa* to numerous antibiotics such as chloramphenicol, nalidixic acid, erythromycin and others [156]. A representative of the isothiocyanates class, sulforaphane showed antibacterial effect on *H. pylori* both in vitro and in vivo [157,158]. Regarding the antibacterial activity of glycosides, Soulef tested an extract of *Glycyrrhiza glabra* root on three bacterial cultures (*E. coli*, *P. aeruginosa and S. aureus*). He observed that the extract was active on *P. aeruginosa* and *S. aureus*, but no effect was noticed on *E. coli* [159]. Thiosulfinates can have both bacteriostatic and bactericidal effects. A major representative of the thiosulfinates class is allicin (diallylthiosulfinate) obtained from garlic (*Allium sativum L*.). The mechanism of action of allicin consists of binding to thiol groups and inactivation of essential enzymes in bacteria. It is active at µM concentrations on Gram-positive and -negative bacteria, including those resistant to antibiotics, and fungi [160]. Table 2 summarizes the main plants with antibacterial action together with their chemical composition and the spectrum of action.

## 6. Antibiotics and Microbiota

The human intestine is the habitat for a diverse community of microorganisms consisting of bacteria, archaea, eukaryotes and viruses [170]. Bacteria represent the majority of the microorganisms that compose the human gut microbiota, with over 1000 species, most of which are anaerobes [173,174].

About 90% of the bacteria present in human feces and intestinal mucosa belong to two phyla, *Bacteroidetes* and *Firmicutes*, while the other species identified in low abundance are members of *Actinobacteria* and *Proteobacteria* phyla [175].

The intestinal microbiota is a vital part of the body, playing numerous roles, from the synthesis of compounds to actions in the metabolism of endo- and exogenous substances [176]. Bacterial diversity of the microbiota can influence the proper functioning of the body and the appearance of pathologies [177].

The interaction between drugs and gut microbiota is relevant for understanding drugs’ mechanisms and the occurrence of certain drug side effects [178].

Antibiotics are used worldwide at an enormous scale, being some of the most prescribed drugs. Despite their benefits, antibiotics are potentially harmful agents, noting the link between their excessive use and the development of many disorders associated with the alteration of the intestinal microbiota [179,180].

Antibiotics are used as prophylactic and curative treatment for a wide range of life-threatening bacterial infections, so countless lives have been saved by antibiotics. In recent years, studies conducted on the microbiota have shown that several factors related to antibiotics, namely class, pharmacokinetics, pharmacodynamics, dose, duration and route of administration, can lead to undesirable consequences [175,181].

The broad spectrum of action of most antibiotics available on the market determines a toxic impact not only on harmful bacteria but also on healthy ones. The damage to the intestinal microbiota is the main effect of antibiotics on the gut, while other mechanisms of action have also been described: a cytotoxic effect on epithelia and the dissemination of antibiotic-resistant microorganisms [182].

Antibiotics exhibit different effects and consequences on the gut microbiota due to their different spectrum of activity and bacterial target, as presented in Table 3.

Lincosamides, and, in particular, clindamycin, a broad-spectrum antibiotic with preponderant biliary elimination and high concentrations in stools, represent one of the most critical risk factors for the initiation of *Clostridium difficile* infection [183]. 

A short exposure to clindamycin led to long-term disorders of microbiota composition, characterized by a reduction in the clonal diversity of *Bacteroides* and an augmentation in the levels of specific resistance genes [193]. Moreover, clindamycin treatment affected Gram-positive aerobic and anaerobic bacteria, after administration for 10 days to healthy humans, together with a long-term impairment of microbiota [185].

Another class of antibiotics, macrolides, more precisely clarithromycin, caused a sustained decrease of *Actinobacteria* observed in *Helicobacter pylori*-positive patients under standard therapy [186].

In a large cohort of Finnish children, it was observed that macrolides induced long-term alterations of microbiota, mainly by decreasing the number of *Actinobacteria and Firmicutes* and enhancing the relative abundance of *Bacteroidetes* and *Proteobacteria*, while molecules of the β-lactam class were not associated with significant changes in the microbiota composition [187].

Intrapartum antibiotic prophylaxis that included penicillin, ampicillin or ampicillin in combination with erythromycin was associated with decreased levels of *Firmicutes* and *Actinobacteria* and an increase in *Proteobacteria* [188].

In addition, the combination of ampicillin and cephalosporins determined a decrease in *Firmicutes* and an increment in *Proteobacteria* and *Bacteroidetes* together with a reduced microbial diversity, whereas fluoroquinolones increased *Bacteroidetes* and *Proteobacteria*, without modifying *Firmicutes* [189].

It has also been reported that fluoroquinolones, such as ciprofloxacin, act against Gram-negative facultative anaerobe bacteria, while levofloxacin reduces the number of Gram-positive anaerobes, including *Bifidobacteria* [190].

Vancomycin in combination with bacitracin decreased *Bacteroidetes* and *Firmicutes* as well as the total microbiota richness after administration to C57BL/6J mice [191]. In addition, in a randomized controlled study in obese people, oral vancomycin reduced bacterial diversity and *Firmicutes* while enhancing *Proteobacteria* [192].

Some antibiotics have stimulated the growth of beneficial bacteria, having a positive effect, i.e., exerting a so-called “eubiotic” effect. An example is nitrofurantoin, which, when administered to patients suffering from urinary tract infections, demonstrated an increase in *Actinobacteria*, mainly *Bifidobacteria*, as well as *Fecalibacterium* genera [193,194].

Increasing evidence asserts the “eubiotic” properties of rifaximin, a nonabsorbable, oral antibiotic derived from rifamycin. Thus, in a trial conducted on patients with irritable bowel syndrome, the antibiotic augmented the bacterial diversity and the *Firmicutes*/*Bacteroidetes* ratio, while, in another study on patients with diverse gastrointestinal and liver disorders, rifaximin increased the abundance of *Lactobacilli*, with no effects on bacterial diversity [195,196].

Antibiotics have a significant capacity to influence the gut microbiota composition, leading to consequent clinical manifestations, by inducing either a “dysbiotic” or “eubiotic” effect.

In these circumstances, the modulation of gut microbiota should be considered a novel therapeutic target not only for infectious diseases but also for all other disorders associated with an impairment of intestinal microbiota.

A reasonable use of antibiotics in clinical practice s recommended for a better management of patients with disorders related to the impairment of intestinal microbiota.

## 7. Antibiotics and Cancer

Although cancer is a widespread disease around the world today, it still raises concern regarding the treatment and the survival rate of patients. In 2020, over 19 million new cases of cancer were recorded, as well as almost 10 million cancer-related deaths [197]. Modern cell biology tries to explain the basic mechanisms underlying cancer. These mechanisms can be summarized as follows: (i) abnormal cell growth; (ii) migration of cells with uncontrolled cell cycle; and (iii) continuous recovery and reproduction of cancer stem cells [198]. Current cancer therapy consists of surgery, radiation, chemotherapy, immunotherapy and targeted therapy. Surgery is used to treat localized cancers that have not metastasized. Other therapies can be used to treat systemic cancer, including metastases [199]. 

The discovery of antibiotics was the starting point of modern medicine. We have since witnessed an exponential increase in antibiotics use globally. Between 2000 and 2015, antibiotics consumption increased by 65% [200]. Recent research in the field of anticancer therapy and antibiotics showed that some antibiotics can cause apoptosis of cancer cells, thus preventing their growth and metastasis [201]. On the other hand, other studies have mentioned that the consumption of antibiotics can cause a disturbance of the saprophytic microbial flora. The intestinal microbiome plays an important role in the treatment of cancer. Thus, the consumption of antibiotics can cause, in addition to the destruction of the intestinal flora, a decrease in the immune system and a promotion of the inflammatory processes, all of which have pro-cancer effects and lead to a reduction in the effectiveness of cancer treatment [202]. Thus, antibiotics can be viewed as a two-edged sword, exerting a beneficial effect in cancer therapy as well as a pro-cancer effect, representing a risk to humans.

### 7.1. Antibiotics as Cancer Therapy 

Along with the development of biomedicine and technology, the cancer process became better understood. A brief definition of cancer from the perspective of cell biology could be that cancer is a cellular pathology characterized by abnormal cell growth. Antibiotics with antitumor effect are one of the most important classes of antibiotics. The antitumor mechanism of action is mainly based on the following approaches: (i) the anti-proliferative effect; (ii) the anti-apoptotic effect; and (iii) the inhibition of epithelial–mesenchymal transition (EMT) [203].

Anticancer antibiotics are mainly anthraquinones and peptides that determine an inhibitory effect on the proliferation and uncontrolled growth of cancer cells. The main classes of antibiotics with effect on cancer cells are anthracyclines, bleomycin, actinomycin, mitomycin, guanorycin and endiyne [204]. 

The anticancer effects of antibiotics are varied, acting on many cancers of different origin. Doxorubicin, member of the anthraquinone class, is an example of a broad-spectrum antibiotic that proved to be a potent anticancer agent. Studies have shown that it is effective in treating solid tumors such as breast cancer, lung cancer and liver cancer, but it also has beneficial effects on acute leukemia [205,206]. The mechanism of action of doxorubicin is based on the insertion of the anthracyclic plane between the DNA base rods, thus affecting the spatial structure of DNA and inhibiting DNA and DNA-dependent RNA synthesis. It also acts selectively on purine nitrogenous bases [207]. In addition, Al-Aamri et al. observed in an in vitro study that doxorubicin can inhibit the activity of nuclear topoisomerase, causing the breakdown of double- and single-stranded DNA [208]. Mitomycin has been studied in vivo for its antitumor effects in liver cancer and metastatic breast cancer. The advantage of mitomycin is the very fast healing effect; however, it has a very high toxicity [209]. Mitomycin acts by inhibiting the DNA replication and synthesis forming double- or intra-strand cross-links with DNA [210]. Another example of an antibiotic with a proven antitumor effect is epirubicin. The effects of epirubicin on different types of cancer have been studied, and its antitumor effect has been observed in breast cancer [211], esophago-gastric cancer [212], melanoma [213], colon cancer [214] and ovarian cancer [215], among other types of cancer. The antitumor mechanism of action of epirubicin is based on its ability to intercalate between nucleotides, thus altering the transcription process and blocking mRNA formation and DNA and RNA synthesis. It also exerts an inhibitory effect on topoisomerase II [216]. Other examples of antibiotics with proven antitumor effect are presented in Table 4.

**Figure 10:**
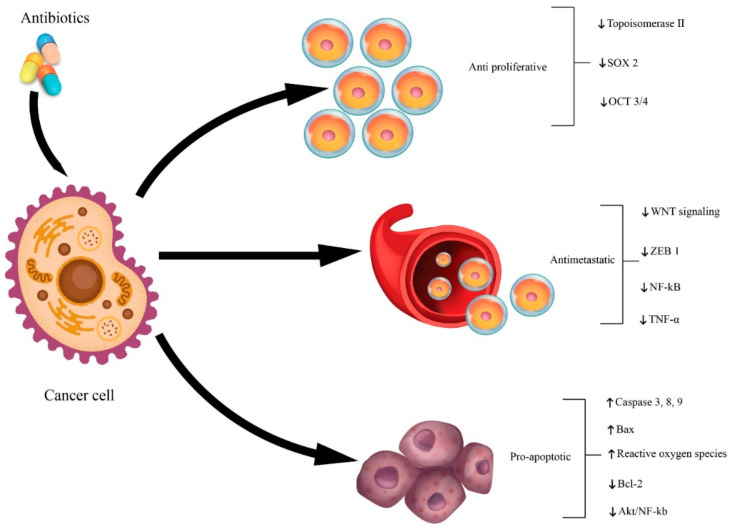
Summary of the antitumor mechanisms of action of antibiotics.

The antitumor activity of antibiotics relies on three main mechanisms (see Figure 10): (i) antiproliferative effect; (ii) pro-apoptotic effect; and (iii) anti-epithelial-to-mesenchymal transition (EMT) effect. The antiproliferative effect is exerted by affecting the cell cycle. Antibiotics can affect the entire proliferative cycle, including the G0 phase. An example is cyclinenone-specific drugs [230]. The pro-apoptotic effect is due to the ability of antitumor antibiotics to act on proapoptotic B cell lymphoma-2 (Bcl-2) genes, apoptotic pro-Bcl-2 associated x (Bax), caspase-3/8/9 and cancer suppressor gene p53. An example of an antibiotic that acts as a pro-apoptotic drug is salinomycin [231]. To limit metastasis, some antibiotics such as salinomycin may inhibit proliferation by acting as an EMT regulatory agent [232]. 

Recent studies have shown that cancer cells are increasingly resistant to conventional antitumor antibiotics while being extremely sensitive to classes of anticancer antibiotics to which they have never been exposed. For this reason, antibiotics such as doxorubicin or mitomycin have begun to be replaced by more recent antitumor antibiotics such as salinomycin, 25driamycin or fluoroquinolones. Studies in the field have gained great interest due to the effectiveness of these antibiotics [222].

### 7.2. Antibiotics as Cancer Promoters

Although antibiotics are considered the cornerstone of modern medicine, recent studies have evidenced that these compounds can alter patients’ health by altering the microbial flora. Disruption of the intestinal microbiome is associated with various systemic pathologies. Intestinal dysmicrobism represents a specific feature not only for intestinal diseases but also for extra-intestinal diseases such as neurological or metabolic disorders [233].

Oncological studies have pointed out that some neoplasms can occur due to the alteration of the microbiome. For example, colorectal cancer can be caused by altered fecal saprophytic bacterial flora, among other factors [234]. It seems that *Fusobacterium* spp. plays an important role in the pathogenicity of colorectal cancer through an inflammatory mediated mechanism, but the mechanism of action requires additional clarification [235].

### 7.3. Potential Pro-Tumoral Mechanisms of Action Related to Antibiotics

A direct carcinogenic effect of antibiotics has not been established yet. The main hypothesis regarding their pro-tumoral effect refers to their influence on the human microbiome [236]. Numerous relevant publications in the field have raised the issue of antibiotic use and its association with cancer. According to a recent meta-analysis of 25 observational studies, it has been shown that prolonged and excessive consumption of antibiotics throughout lifetime can increase the risk of developing various types of cancer [237].

In the meta-analysis performed by Petrelli et al. [237], approximately 8,000,000 individuals were studied. A lifelong exposure to antibiotics was correlated with an increase of cancer risk by 18% compared to individuals who had never used antibiotics. The types of cancer associated with antibiotics use included lung cancer, pancreatic cancer, multiple myeloma, kidney cancer and lymphomas. The people at highest risk were those who were exposed to high doses of antibiotics for a long time. In addition, the antibiotics with the highest carcinogenic risk were β-lactams, quinolones and macrolides [237].

Moreover, the results of studies that investigated the association between the local microbiota and the onset of cancer indicate that certain types of lung cancer can occur due to an altered lung microbiota. It has been observed that, in patients with lung cancer, bacterial species that are part of the lung microbiota were found in greater numbers [238]. In addition, in the aforementioned meta-analysis, a correlation was made between lung cancer and antibiotic consumption, which can be explained by the modification of the lung microbiota [237].

Until recently, the urinary tract was considered a sterile environment. The role of the urinary microbiota on the patients’ health status is currently under study. Microorganisms found at this level can have both beneficial and harmful roles. Currently, increasing evidence indicates that bacteria in the urinary microbiota help to prevent bladder cancer. By consuming antibiotics and altering the urinary microbiota, there may be an increased risk of bladder cancer [239]. A link between the immune system and the intestinal microbiota has also been observed with the appearance of lymphoma associated with the mucosa [240].

The role of antibiotics in the pathogenicity of cancer is debatable. While some studies show that antibiotics have a beneficial effect on various types of cancer, other studies show their devastating effect on health. Excessive consumption of antibiotics and alteration of the intestinal microbiome play important roles in the occurrence of various types of cancer. For these reasons, antibiotic use should be limited as much as possible, and physicians should intervene in educating the population about irrational antibiotic use.

## 8. Conclusions and Future Perspective

Even though for a long time infectious diseases were kept under control by antibiotic therapy, the emergence of resistant bacteria opened a new gap that has become exceedingly difficult to cover at present. The limitations of the current antibiotic therapy, namely severe adverse effects and the increased incidence of resistant bacteria to conventional antibiotics, require stringent action. Important steps have been taken in this direction, unraveling the mechanisms of action responsible for the antibacterial effect, as well as for the side effects and bacterial resistance, thanks to advancement in the field of cellular and molecular biology and in the technological domain, but these issues are not completely elucidated. A better comprehension of the antibiotics’ mode of action together with adequate prescription and administration could reduce antibiotic toxicity and resistance.

Finding new therapeutic alternatives to conventional antibiotherapy is attracting increased interest among scientists, natural compounds representing the main subject of pursuit nowadays. The main advantages of these compounds consist in the different mechanisms of action that can overcome bacterial resistance and the reduced side effects. Albeit significant progress has been recorded in this direction, further studies are needed to fully elucidate their antibacterial mechanisms of action and verify their potential synergistic effects in combination with conventional antibiotics as a novel efficient alternative to combat bacterial resistance.

The link between antibiotics and microbiota and its implications in cancer development/arrest is a subject of novelty that needs further elucidation. A correct characterization and pertinent understanding of antibiotics’ complex mode of action (as antibacterial and antitumor/pro-tumoral agent) require many resources and continuous research that will expand our knowledge and offer the answers to win the fight against these debilitating diseases (infectious diseases and cancer).

## Figures and Tables

**Table 1:** Antibiotics’ classification by the mechanism of action.

Antibiotic Class		Target Site	Representatives
**β-lactams**	Penicillins	Cell wall synthesis	Penicillin G and Penicillin V, Methicillin, Oxacillin, Cloxacillin, Dicloxacillin, Nafcillin, Ampicillin, Amoxicillin, Carbenicillin, Ticarcillin, Mezlocillin, Piperacillin, Azlocillin, Temocillin
	Cephalosporins	Cell wall synthesis	**1st generation**: Cephalothin, Cephapirin, Cephradine, Cephaloridine, Cefazolin **2nd generation**: Cefamandole, Cefuroxime, Cephalexin, Cefprozil, Cefaclor, Loracarbef, Cefoxitin, Cefmetazole **3rd generation**: Cefotaxime, Ceftizoxime, Ceftriaxone, Cefoperazone, Ceftazidime, Cefixime, Cefpodoxime, Ceftibuten, Cefdinir **4th generation**: Cefpirome, Cefepime **5th generation**: Ceftaroline, Ceftobiprole
	Carbapenems	Cell wall synthesis	Imipenem, Meropenem, Doripenem
	Monobactams	Cell wall synthesis	Aztreonam
**Macrolides**		Protein synthesis inhibitors—Inhibit 50 s subunit	Erythromycin, Azithromycin, Clarithromycin
**Tetracyclines**		Protein synthesis inhibitors—Inhibit 30 s subunit	Tetracycline, Chlortetracycline, Oxytetracycline, Demeclocycline, Minocycline, Methacycline, Doxycycline, Tigecycline
**Aminoglycosides**		Protein synthesis inhibitors—Inhibit 30 s subunit	Streptomycin, Neomycin, Kanamycin, Paromomycin, Gentamicin, Tobramycin, Amikacin, Netilmicin, Spectinomycin, Sisomicin, Isepamicin
**Sulfonamides**		Folic acid synthesis inhibitors	Prontosil, Sulfonamide, Sulfanilamid, Para-Aminobenzoic Acid, Sulfadiazine, Sulfisoxazole, Sulfamethoxazole, Sulfathalidine
**Quinolones**		DNA synthesis inhibitors	Nalidixic Acid, Ciprofloxacin, Norfloxacin, Pefloxacin, Enoxacin, Ofloxacin, Levofloxacin, Sparfloxacin, Lomefloxacin, Fleroxacin
**Izoniazid**		Mycolic acid synthesis inhibitors	
**Ansamycin**		RNA synthesis inhibitors	Rifampin
**Polymycins**		Cytoplasmic membrane structure	
**Daptomycin**		Cytoplasmic membrane structure	

**Table 2:** Plants with antimicrobial activity.

*Scientific Name* (Common Name)	Chemical Constituents	Spectrum of Action	References
*Aleurites moluccanus* (Candlenut)	3-acetyl aleuritolic acid, moluccanin	*C. albicans*, *S. aureus*, *E. faecalis*	[161]
*Aloe barbadensis* (Aloe vera)	Reducing sugar, tannins, anthraquinone, glycoside, saponins, flavonoids	*S. aureus MRSA.*, *K. pneumonia*	[162]
*Azadirachta indica* (Neem)	Reducing sugar, tannins, glycoside, saponins, flavonoids, steroids, terpenoids	*S. aureus*, *E. coli*	[162]
*Bergenia ciliata*	Tannins, terpenoids, alkaloids, flavonoids	*S. aureus*, *B. subtilis*, *P. vulgaris*, *P. aeruginosa*, *E. coli*	[163]
*Buchenavia* *Tomentosa* (Merindiba)	Ellagic acid, gallic acid, myricetin-3-O-rhamnopyranoside, quinic acid	*E. coli*, *P. aeruginosa*, *S. aureus*, *S. epidemidis*	[164]
*Bryophyllum pinnatum* (Bryophyllum)	reducing sugar, tannins, glycoside, saponins, flavonoids, steroids, terpenoids	*E. coli*, *S. aureus*, *P. mirabilis*	[162]
*Cannabis sativa*	α-Pinene, myrcene, linalool, limonene, trans-β-ocimene, α-terpinolene, trans-caryophyllene, α-humulene, caryophyllene	*B. cereus*, *E. coli*, *B. subtilis*, *S. typhi*	[165]
*Caryophyllus aromaticus* (Clove)	Essential oils (eugenol), tannins and flavonoids	*S. aureus*, *S. choleraesuis*, *P. aeruginosa*, *C. albicans*, *K. pneumoniae*, *K. pneumonia*, *Shigella* spp., *Proteus* spp., *P. aeruginosa*	[166]
*Crataegus azarolus var. aronia calli* (Hawthorn)	Pro-anthocyanidins, flavonoid, (−)-epicatechin, procyanidin B2, chlorogenic acid and hyperoside	*Staphylococcus aureus*, *S. epidermidis*, *M. luteus*	[167]
*Croton doctoris*	Malic acid, scopoletin, ferulic acid, ent-18-hydroxy-trachyloban-3-one, crotonin, cajucarin B	*A. naeslundii *, *L.acidophilus*, *S. gordonii*, *S. mutans*, *S. sanguinis*, *S. sobrinus*, *S. mitis*	[168]
*Croton urucurana* (Baillon)	Borneol, bornyl acetate, 1-isopropyl-7-methyl-4-methylene-1,3,4,5,6,8-hexahydro-2H-naphthalen-4a-ol, sesquicineole g-gurjunene epoxide	*S. aureus*, *S. epidermidis*, *P. aeruginosa*, *E. coli*, *S. setubal*, *K. pneumoniae*, *S. cerevisiae*, *C. neoformans*	[169]
*Curcuma longa* (Turmeric)	Carbohydrates, saponins, flavonoids, tannins	*S. pyogenes*, *S. aureus*, *P. aeruginosa*	[170]
*Commiphora molmol* (Myrrh)	Carbohydrates, alkaloids, saponins	*S. pyogenes*, *S. aureus*, *P. aeruginosa*	[170]
*Cymbopogon citratus* (Lemongrass)	Tannins, glycoside, saponins, flavonoids, terpenoids	*S. aureus MRSA.*, *E. coli*, *K. pneumoniae*	[162]
*Dodonaea viscosa* (Hopbush)	Terpenes, phenols, flavonoids, saponins	*B. cereus*, *E.coli*, *S. typhi*	[165]
*Euphorbia helioscopia* (Sun spurge)	Alkaloids, tannins, flavonoids, steroids	*E. coli*, *B. subtilis*, *T. harzianum*, *S. typhi*, *K. pneumonia*, *S. aureus*, *B. subtilis*	[171]
*Jasminum officinale* (Jasmine)	Tannins, terpenoids, alkaloids, flavonoids	*S. aureus*, *B. subtilis*, *P. aeruginosa*, *E.coli*	[163]
*Melissa offficinalis* (Lemon-balm)	Essential oils (containing citral and citronellal monoterpenes), flavonoids and rosmarinic, caffeic and chlorogenic acids	*S. aureus*, *S. choleraesuis*, *K. pneumoniae*	[166]
*Ocimun basilicum* (Basil)	Essential oils (linalool, estragol and eugenol), tannins and flavonoids	*P. aeruginosa*	[166]
*Ocimum sanctum* (Tulsi)	Tannins, glycoside, saponins, flavonoids, steroids, terpenoids	*S. aureus MRSA.*, *E. coli*	[166]
*Orthosiphon aristatus* (Blume)	Diterpenoids, polymethoxylated flavonoids, caffeic acid derivatives	*C. albicans*, *S. aureus*, *E. faecalis*, *P. aeruginosa*	[161]
*Origanum vulgare* (Oregano)	Reducing sugar, tannins, glycoside, saponins	*S. aureus MRSA.*, *Bacillus amyloliquefaciens*, *B. brevis*, *B. subtilis*, *S. aureus*, *C. xerosis*, *E. coli*, *K. pneumoniae*, *P. vulgaris*, *M. smegmatis*	[162]
*Pimpinella anisum* (Anise)	Carbohydrates, saponins, flavonoids	*E.coli*, *P. aeruginosa*	[170]
*Psidium guajava* (Guava)	comarins, essential oils, flavonoids, triterpenes and ellagitannins	*S. aureus*, *C. albicans*	[166]
*Punica granatum* (Pomegranate)	Ellagitannins and alkaloids	*P. aeruginosa*, *B. subtilis*	[166]
*Rosmarinus officinalis* (Rosemary)	Flavonoids, phenolic acids (caffeic, chorogenic and rosmarinic) and essential oils (camphor and cineole) and diterpenes	*B. subtilis*, *C. albicans*, *S. aureus MRSA.*, *E. coli*, *P. mirabilis*, *K. pneumoniae*, *P. aeruginosa*	[162,166]
*Santalum album* (Indian sandalwood)	Tannins, terpenoids, alkaloids, flavonoids	*S. aureus*, *B. subtilis*, *P. vulgaris*, *E. coli*	[163]
*Solanum nigrum* (Black nightshade)	alkaloids, cardiac glycosides, flavonoids, saponins, tannins, volatile oils	*B. cereus*, *E. coli*, *P. aeruginosa*, S. typhi	[165]
*Syzygyum joabolanum* (Jambolan)	Flavonoids and tannins	*S. aureus*, *C. albicans*, *Proteus* spp., *K. pneumonia*, *Proteus* spp., *P. aeruginosa*, *E. aerogenes*, *S. aureus*	[166]
*Thymus vulgaris* (Thyme)	Essential oils (mainly thymol and carvacrol), flavonoids, tannins and triterpenes	*S. aureus*, *C. albicans*, *Proteus* spp., *P. aeruginosa*, *E. coli*, *S. aureus MRSA.*, *P. mirabilis*, *K. pneumoniae*	[162,166]
*Vitis vinifera* (Grape)	Gallic acid	*E. coli*, *S.s aureus*	[172]
*Woodfordia floribunda* (Fire flame)	Diethyl phthalate, thymol, woodfordin C	*C. albicans*, *S. aureus*, *E. faecalis*	[161]
*Zingiber officinale* (Ginger)	Carbohydrates, alkaloids, steroids, saponins	*S. pyogenes*, *S. aureus*, *P. aeruginosa*	[170]

**Table 3:** The effects of different antibiotics on gut microbiota.

Antibiotic Representative	Effects on Intestinal Microbiota	Ref.
Clindamycin	↓ Bacteroides diversity	[184]
	↓ Gram-positive aerobic and anaerobic bacteria	[185]
Clarithromycin	↓ *Actinobacteria*	[186,187]
	↓ *Firmicutes* ↑ *Proteobacteria* ↑ *Bacteroidetes*	[187]
Erythromycin	↓ *Actinobacteria* ↓ *Firmicutes* ↑ *Proteobacteria*	[188]
Penicillin Ampicillin	↓ *Actinobacteria* ↓ *Firmicutes* ↑ *Proteobacteria*	[188,189]
	↑ *Bacteroidetes*	[189]
Cephalosporins	↓ *Firmicutes* ↑ *Bacteroidetes* ↑ *Proteobacteria*	[189]
Fluoroquinolones Ciprofloxacin, Levofloxacin	↑ *Proteobacteria* ↑ *Bacteroidetes*	[189]
	↓Gram-negative facultative anaerobes ↓Gram-positive anaerobes ↓ *Bifidobacteria*	[190]
Vancomycin	↓Total bacterial diversity ↓ *Firmicutes* ↓ *Bacteroidetes*	[191,192]
	↑ *Proteobacteria*	[192]
Bacitracin	↓ *Firmicutes* ↓ *Bacteroidetes* ↓Total bacterial diversity	[191]
Nitrofurantoin	↑ *Actinobacteria* ↑ *Bifidobacteria* ↑ *Fecalibacterium* genus	[193,194]
Rifaximin	↑ Bacterial diversity ↑ *Firmicutes/Bacteroidetes* ratio	[195]
	↑ abundance of *Lactobacilli*	[196]

Note: The meaning of the symbols: ↑—stimulation of bacterial culture; *↓*—inhibition of bacterial culture.

**Table 4:** Examples of antibiotics with antitumor effect and their known mechanisms of action.

Antibiotics	Type of Cancer	Mechanism of Action	Ref
Adriamycin	Malignant lymphoma, breast cancer, lung cancer	- Inhibition of the activity of topoisomerase I and II causing the breakdown of DNA with two strands - Upregulation of p53 - Alteration of the Bcl-2/Bax pathway (changing the Bcl-2/Bax ratio)	[217]
Bleomycin	Lymphomas, germ cell tumors, melanomas, gynecologic cancers	- DNA binding - Production of free radicals (hydroxyl radicals)	[218,219]
Ciprofloxacin	Bladder cancer, colorectal cancer, hepatocellular carcinoma, melanoma, breast cancer	- Inhibition of the activity of topoisomerase II - Impairment of cell cycle at the G2 / M checkpoint	[220,221]
Dactinomycin	Nephroblastoma, chorionic epithelial carcinoma, rhabdomyosarcoma and neuroblastoma	- Specific interaction with deoxyguanine in DNA - Formation of complexes with DNA - Inhibition of the mRNA synthesis	[222,223]
Daunorubicin	Acute myelogenous leukemia, lymphocytic leukemia	- Chimerism between basic DNA pairs - Tight DNA binding - Obstruction of the spatial structure of DNA	[224]
Epirubicin	Breast cancer, malignant lymphoma, soft tissue sarcoma, gastric cancer, malignant melanoma, colon cancer, lung cancer, ovarian cancer	- Direct incorporation between DNA nucleotide pairs - Interfering with the transcription process and inhibiting mRNA formation - Topoisomerase II inhibition	[216]
Gemifloxacin	Breast cancer	- Inhibition of bacterial DNA gyrase and topoisomerase IV - Suppression of activity of NF-kB - Inhibition of the migration and invasion induced by cancer necrosis factor (TNF-α).	[225,226]
Mitomycin	Bladder cancer	- Formation of double cross-links or intrachain with DNA - Inhibition of DNA replication and synthesis - Increase in oxygen radicals	[227,228]
Plicamycin	Testicular embryonal cancer, glioma, lymphoma	- DNA binding - Inhibition of RNA synthesis - Acting on cell proliferation at all stages	[229]

## Data Availability

No new data were created or analyzed in this study. Data sharing is not applicable to this article.
